# Investigation of Nucleation and Growth at a Liquid–Liquid Interface by Solvent Exchange and Synchrotron Small-Angle X-Ray Scattering

**DOI:** 10.3389/fchem.2021.593637

**Published:** 2021-07-20

**Authors:** Elyse A. Schriber, Daniel J. Rosenberg, Ryan P. Kelly, Anita Ghodsi, J. Nathan Hohman

**Affiliations:** ^1^ Institute of Materials Science and Department of Chemistry, University of Connecticut, Storrs, CT, United States; ^2^ Molecular Biophysics and Integrated Bioimaging Division, Lawrence Berkeley National Laboratory, Berkeley, CA, United States; ^3^ Biophysics Group, University of California, Berkeley, Berkeley, CA, United States

**Keywords:** mocha, metal-organic chalcogenolate, self-asssembly, hybrid material, mithrene

## Abstract

Hybrid nanomaterials possess complex architectures that are driven by a self-assembly process between an inorganic element and an organic ligand. The properties of these materials can often be tuned by organic ligand variation, or by swapping the inorganic element. This enables the flexible fabrication of tailored hybrid materials with a rich variety of properties for technological applications. Liquid-liquid interfaces are useful for synthesizing these compounds as precursors can be segregated and allowed to interact only at the interface. Although procedurally straightforward, this is a complex reaction in an environment that is not easy to probe. Here, we explore the interfacial crystallization of mithrene, a supramolecular multi-quantum well. This material sandwiches a well-defined silver-chalcogenide layer between layers of organic ligands. Controlling mithrene crystal size and morphology to be useful for applications requires understanding details of its crystal growth, but the specific mechanism for this reaction remain only lightly investigated. We performed a study of mithrene crystallization at an oil-water interfaces to elucidate how the interfacial free energy affects nucleation and growth. We exchanged the oil solvent on the basis of solvent viscosity and surface tension, modifying the dynamic contact angle and interfacial free energy. We isolated and characterized the reaction byproducts via scanning electron microscopy (SEM). We also developed a high-throughput small angle X-ray scattering (SAXS) technique to measure crystallization at short reaction timescales (minutes). Our results showed that modifying interfacial surface energy affects both the reaction kinetics and product size homogeneity and yield. Our SAXS measurements reveal the onset of crystallinity after only 15 min. These results provide a template for exploring directed synthesis of complex materials via experimental methods.

## Introduction

**FIGURE 1:**
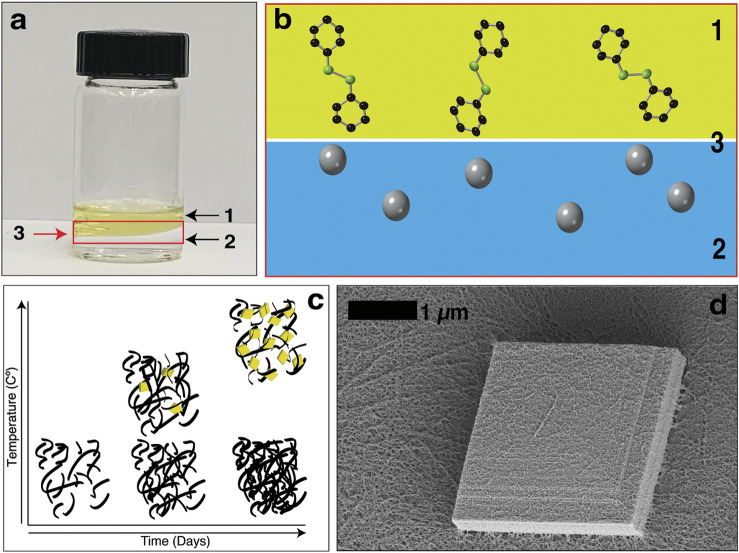
**(A)** An image of the interfacial synthesis of mithrene. Where (1) is the diphenyl diselenide in toluene layer, (2) is the aqueous silver nitrate layer, and (3) is the oil-water interface. **(B)** Diphenyl diselenide molecules and the silver ions in the aqueous solution at the interface. **(C)** A graph showing that temperature control during the interfacial synthesis can be used to isolate the kinetic and thermodynamic products. **(D)** Scanning electron micrograph of a single mithrene microcrystal, where the layered structure is evident, and the amorphous polymer presence is on the surface of the microcrystal.

The metal-organic chalcogenolates (MOCHas) of coinage metals (Cu, Ag, Au) and simple organic ligands have attracted interest as a class of hybrid nanostructured materials (Dance et al., 1984; Dance and Fisher, 1994; Yeung et al., 2020; Hu et al., 2009; Laibinis et al., 1991; Lavenn et al., 2016) with interesting optoelectronic properties (Veselska et al., 2019; Yan et al., 2017; Mak et al., 2010), mechanochemistry (Jodlowski et al., 2016; Yan et al., 2018), and intrinsic chemical tunability (Cheng et al., 2014; Kumar et al., 2013). These compounds have properties reminiscent of monolayer materials but express them in their bulk state. Mithrene, silver (I) benzeneselenolate, is a well understood example of this material class. It is a direct gap semiconductor with a large exciton binding energy, that exhibits optical properties similar to transition metal dichalcogenide monolayers like MoS_2_ (Chen et al., 2017; Trang et al., 2018; Maserati et al., 2020; Yao et al., 2020). A classic synthetic method for mithrene is nucleation and growth occurring at an oil-water interface, in Figure 1A an image of the toluene-water interface and a schematic in Figure 1B help illustrate this synthetic environment.

The liquid-liquid interface offers a unique phase boundary for the formation of solid materials, including crystalline materials (Gautam et al., 2004) and low-dimensional organic materials like graphene (Biswas and Drzal, 2009). Biphasic synthesis can be used to grow large (>3 µm) rhombic single crystals, providing a useful way to access complex materials via a straightforward synthetic approach. In our earlier studies, we examined the role of reagent concentration and temperature on its interfacial crystallization (Schriber et al., 2018; Forster et al., 2002) and their role in the assembly of both the target crystals and an amorphous byproduct (Schriber et al., 2018; Popple et al., 2018). These studies focused on modifying external components to the interfacial synthesis, as opposed to affecting the interface itself.

Here, we took inspiration from heterogeneous nucleation studies of aerosol and cloud drop formation at fluid interfaces in the atmosphere (Hienola et al., 2007) and focused our studies on modifying the liquid-liquid interface of this synthesis through solvent exchange. By switching out the organic solvent based on solvent viscosity and surface tension, we were able to establish that changes to the interfacial free energy and even inversion of the organic and aqueous component changes the reaction kinetics and the nucleation and growth process of mithrene. Using temperature reduction, we were able to isolate and identify the amorphous biproduct as the kinetic product, and mithrene as the thermodynamic product of this synthesis. In Figure 1C, the trend between temperature increase and decrease can be seen in a graphical image, with temperature on the *y*-axis and incubation time no the *x*-axis. Furthermore, using synchrotron small-angle X-ray scattering, we were able to observe the presence of crystalline mithrene 15 min into the synthesis, which far surpasses the time-resolution of methods like SEM.

## Methods and Materials

### Materials

Silver nitrate (>99%) and diphenyl diselenide (98%) were used as received by Sigma-Aldrich (St. Louis, MO) and TCI America (Portland, OR). Toluene was used as received by EMD Millipore (Hayward, CA). 18.2 MΩ deionized water was supplied by Millipore (Billerica, MA). Glass scintillation vials and Thermo-Fast™ 96-Well Full-Skirted Plates were purchased from Thomas Scientific (24-400). Plastic conical 1.5 ml tubes were purchased from Thomas Scientific. Dichloromethane (99.6%) was used as received by Acros Organics. DMSO (99.7%) was used as received by Cambridge Isotope Labs Incorporated. Ethyl acetate (<99.5%) was used as received by Fisher Scientific. Cyclohexane (≥99%) was used as received by Avantor.

### Preparation of Silver Benzeneselenolate (Mithrene)

Mithrene is prepared via crystallization at an immiscible liquid-liquid interface, as previously reported (Schriber et al., 2018). Volumetric 3.0 mM solutions of diphenyl diselenide (DPSe) and silver nitrate were prepared in toluene and water, respectively. Immiscible interfaces were obtained by first pipetting 3.0 ml of the aqueous silver nitrate into a 20 ml glass vial. 3 ml of the DPSe solution is gently pipetted onto the aqueous solution. Solutions were capped and allowed to rest on the benchtop for timed intervals. Samples were exposed to normal room lighting.

For the solvent exchange experiments, diphenyl diselenide was suspended in dichloromethane, DMSO, cyclohexane, ethyl acetate, and methyl ethyl ketone to make 3 mM stock solutions. These were pipetted into a 4-dram vial, along with 3 mM aqueous silver nitrate, with the less dense solvent layered on top. Each sample was allowed to incubate at room temperature for 3 days prior to imaging of the interface and harvesting of the product. Harvest was completed using two methods. The interface scooping method, angling the vial and scooping the interface with glass substrate that was rinsed in ethanol and allowed to dry and the dropcast method as detailed below for the time-interval SAXS.

To study crystallization at various time intervals, interfaces were prepared in triplicate and the reaction was quenched at each time interval. Reaction quenching was performed by removal of the aqueous silver nitrate layer via careful pipetting using 10 μL pipette and a 1 ml pipette to prevent breakage of the interface into the silver nitrate layer and product loss. The remaining DPSe solutions were swirled in the glass vial. The crystalline product adheres strongly to the glass, and the remaining DPSe solution was removed by decanting. Glass vials were then placed in a vacuum desiccator for 12 h to ensure that all toluene was removed from the reaction products. The reaction products were then suspended by sonicating for 15 s in 50/50 (v/v) D.I. water/ethanol solvent for SAXS measurements. This suspension was then deposited in a plastic 1.5 ml microtubes, selected as the product has poor adhesion to the plastic. Additional brief sonication was sufficient to keep particles in suspension. For growths under 1 h, suspensions were combined from the products of three reactions (Schriber et al., 2018). While we cannot quantify if there is additional reaction between any residual precursors in the samples after preparation for SAXS measurements, SAXS is a contrast technique and each time point profile will overwhelmingly represent scattering contributions from the most prevalent species. Only in the case of ordered crystal growth will Bragg peaks be observed, allowing us to qualitatively analyze the profiles for presence of crystalline mithrene.

For the cold synthesis, 3 mM of aqueous silver nitrate was added to a 4-dram vial prior to the gentle layering of 3 mM diphenyl diselenide in toluene. The sample was place in a refrigerator at 5°C and allowed to incubate for 3 days. After the allotted time, the product was harvested using the scooping method (Schriber et al., 2018) using silicon substrate.

### Scanning Electron Microscopy

Samples were imaged on a Nanoscience instruments Phenom ProX scanning electron microscope at accelerating voltages between 5 and 15 kV. Samples were prepared on a glass substrate unless otherwise indicated.

### Transmission Electron Microscopy

Transmission electron microscopy was performed on a ZEISS ULTRA-55 field emission scanning electron microscope in scanning transmission electron microscopy mode at accelerating voltages ranging between 15 and 30 kV. Suspended samples of mithrene prepared via the interfacial method for three days were deposited on a Ted Pella lacey carbon grid.

### SAXS Sample Preparation and Data Collection

SAXS data was collected at the SIBYLS beamline (BL12.3.1), at the Advanced Light Source at Lawrence Berkeley National Laboratory, Berkeley, California (Classen et al., 2010). X-ray wavelength was set at λ = 0.127 nm and the sample-to-detector distance was 2010 mm, resulting in scattering vector q, ranging from 0.1 nm^−1^ to 5 nm^−1^. The scattering vector is defined as q = 4πsinθ/λ, where 2θ is the scattering angle. Data was collected using a Dectris PILATUS3X 2M detector at 20°C and processed as previously described (Dyer et al., 2014). Immediately prior to data collection, samples were deposited into 96-well plates, where three 30 μL samples at each time measurement was bracketed with two 30 μL blank 1:1 v/v D.I. water/ethanol samples. Samples are transferred from the 96-well plate to the X-ray beam by a TECAN liquid handling robot as previously reported(Hura et al., 2009; Dyer et al., 2014). Samples were exposed to the X-ray beam for a total of 30 s and scattering images were collected at a frame rate of 0.5 s for a total of 60 images. Two background samples were collected for each sample to reduce error in subtraction and to ensure that the process was not subject to instrument variations. Each collected image was circularly integrated and normalized for beam intensity to generate a 1-dimensional scattering profile by beamline specific software.

### SAXS Data Analysis

The 1-dimensional scattering profile of each sample was background subtracted by each of the two corresponding solvents, producing two sets of background subtracted sample profiles and a third which averaged the two. Profiles were examined for radiation damage. Scattering profiles over the 30 s exposure were sequentially averaged together until radiation damage affects were seen to begin changing the scattering curve. Averaging was performed with web-based software FrameSlice (sibyls.als.lbl.gov/ran). Experimental SAXS profiles were analyzed from averaged 1-D data using SCÅTTER (Hura et al., 2009; Rambo and Tainer, 2013) and the Irena (Ilavsky and Jemian, 2009) SAS macros for Igor Pro (Wavemetrics Inc.).

## Results and Discussion

### Solvent Exchange and Cold Synthesis

Under standard interfacial environment conditions, 3 days of incubation yields ∼1 mg of product total, including both the crystalline mithrene product and the amorphous polymer byproduct. Scanning electron microscopy (SEM) imaging of harvested crystals show significant presence of mithrene crystallites with well-defined edges and standard rhomboidal morphology with the presence of the filamentous polymer. In Figure 1C, a high-resolution TEM image shows the polymer coating the surface of a ∼2 µm mithrene crystallite, where the step edges are well resolved, and the individual strands of the byproduct can be distinguished. Exchanging out the organic solvent resulted in shifting reaction kinetics, mithrene edge morphology, and overall product yield and size as compared to the toluene synthesis.

**FIGURE 2:**
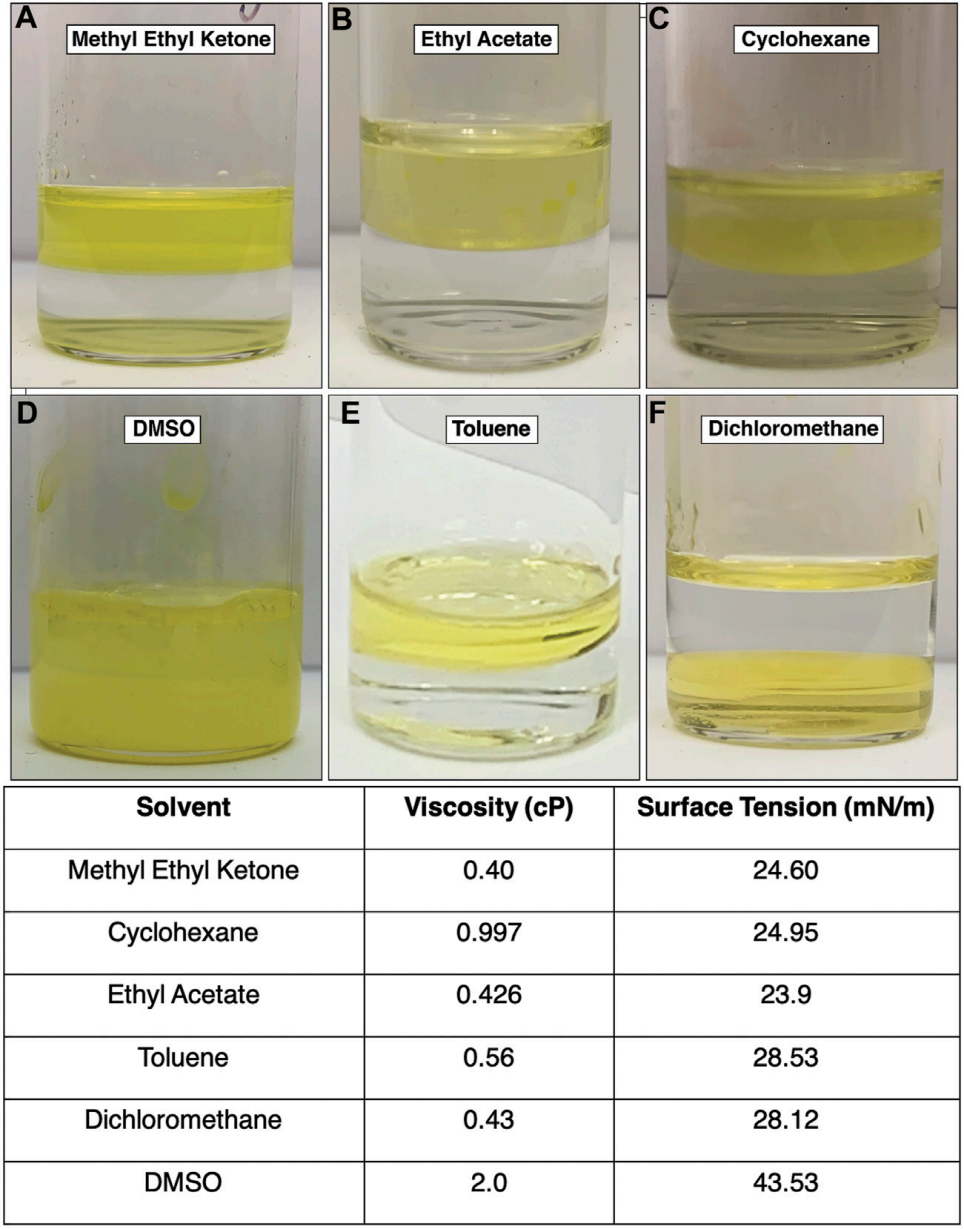
**(A)**–**(F)** Images of each interface using different organic solvents. The dichloromethane interface inverted **(F)** and the DMSO interface broke, with product sinking to the bottom of the vial. The toluene interface is also imaged as a frame of reference. Table 1. Each solvent is listed with its literature viscosity in centipoise (cP), and its literature air-liquid surface tension in millinewtons-per-meter (mN/m).

**FIGURE 3:**
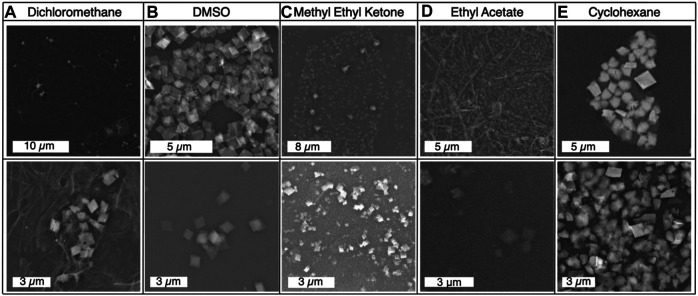
**(A)**–**(E)** Scanning electron micrographs of each product from the solvent exchange syntheses, with products imaged from both dropcasted sample and interface harvested sample using the scooping method.

The density of dichloromethane is higher than that of water. Using this organic solvent required adding the organic solvent first and then layering the aqueous phase. After three days of incubation, some of the aqueous phase had become sandwiched in between a thin layer of organic phase on the surface and the majority of the organic phase still on the bottom. In Figure 2F, this inversion of the interface can be seen after 3 days of incubation. The resultant product was neither rich in the amorphous byproduct or mithrene crystallites, a “pocket” of byproduct and very thin crystallites was observed under the SEM. Figure 3A depicts this pocket of mithrene crystallites with the standard rhomboidal morphology, but very little presence of the polymer byproduct. We attribute this significant loss of product to the fact that only a thin organic phase was present above the aqueous phase, so the lack of diphenyl diselenide precursor to silver nitrate became the limiting factor when harvesting product using the interface scooping method. Using the drop-casting method, where product from the inverted interface was also harvested, also showed minimal presence of crystallites as well, suggesting that inverting the interface changes the interfacial environment enough to reduce product yield and there is synthetic reliance on the organic solvent and precursor being layered on the aqueous phase. DMSO is somewhat miscible in water, therefore, after three days of incubation, diffusion into the aqueous solvent had produced no visible interface, and the majority of the product had aggregated at the bottom of the glass vial. In Figure 2D, the broken interface and aggregation at the bottom can be seen. Even with the broken interface, the DMSO synthesis had the highest yield of mithrene crystallites with the classic rhomboidal shape and sharp edges. These crystals were smaller and more homogenous in size, (∼1 µm) see Figure 3B. This is reminiscent of the mithrene crystals produced using a different solvothermal synthesis with benzeneselenol as the selenium precursor. There was very little presence of the amorphous polymer byproduct. We believe that the slow diffusion of the DMSO into the aqueous layer overtime still allowed for mithrene crystals to form at an interface, but the reducing interfacial surface tension caused the polymer raft to break and the products then sunk to the bottom of the vial.

Methyl ethyl ketone produced an interface with the aqueous layer that had the flattest meniscus, comparative to the other solvents that produced an interface, see Figure 2A. This synthesis yielded very small mithrene crystallites (>500 nm), compared to the toluene synthesis, embedded in the amorphous polymer product. The interface between the ethyl acetate and the aqueous layer had a more curved meniscus, and an exceptionally small quantity of mithrene was recovered, see Figures 2B, 3D for reference. The amorphous polymer was favored, and its diameter was thicker and more rod-like than prior syntheses. Some exceptionally thin mithrene crystals were observed after dropcasting, shown in figure Figure 3D (bottom). The meniscus between cyclohexane and the aqueous layer has the most curvature, shown in Figure 2C. At the interface, there was less mithrene present than DMSO, and these crystals, while stoichiometrically the same as mithrene, had different morphologies. In Figure 3E, there are some thick classic mithrene crystals, surrounded by small immature or misshapen mithrene crystals, the smaller crystals do not have sharp edges and have heterogenous morphologies with both thin and thick areas on individual crystals.

**FIGURE 4:**
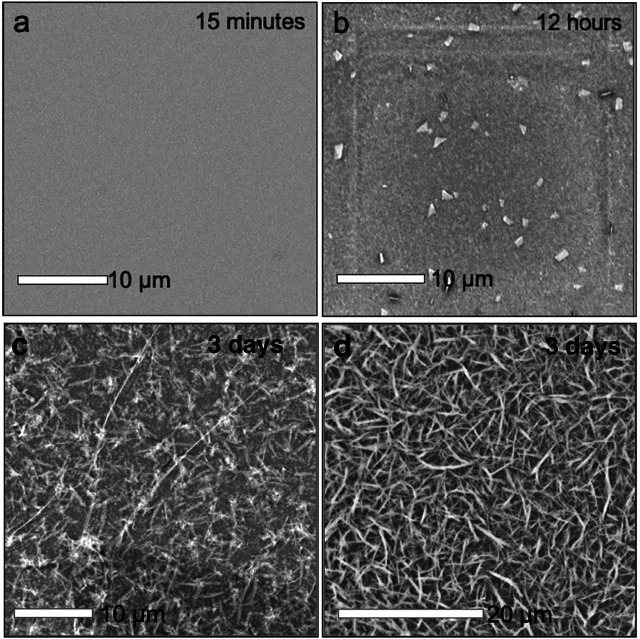
**(A)**–**(D)** Scanning electron micrographs of early stage mithrene synthesis, where at 15 min of growth, samples that were dropcasted onto substrate show no presence of mithrene or polymer product. At 12 h of growth, there are immature crystals present, a result of the quenching the reaction and subsequent dropcasting. In comparison is the cold synthesis, where at three days of incubation at 5°C, there is no mithrene presence and a thick coating of the amorphous polymer product.

The cold synthesis was performed to identify whether the amorphous polymer or the mithrene crystallites were the thermodynamic or kinetic product of the reaction. Under room temperature conditions, after 3 days, there is presence of both mithrene and the amorphous polymer, so differentiating between the two requires removal of energy. After three days of incubation in a 4°C environment, there is no presence of mithrene crystallites, and instead a heavy coating of the amorphous polymer, that adopts a more rigid rod-like morphology. In Figures 4A–D comparison of 12 h of incubation time at room temperature vs. 3 days in 4°C shows that mithrene crystals do not grow in cold temperatures, but the polymer product grows in abundance. This is significant evidence that points toward the mithrene product being the thermodynamic product and the polymer, the kinetic product. It also provides a method for isolating the polymer byproduct.

### Small-Angle X-Ray Scattering of Mithrene

In order to detect the presence of early time-stage synthetic products, a synchrotron-based SAXS source was used (Classen et al., 2013) which minimizes background and produces high intensity scattering. This instrument provided the resolution to detect the presence of the highest intensity (002) diffraction peak of mithrene in the SAXS profiles at extremely low concentrations and early synthetic time points. Unlike diffraction techniques, where interatomic distances between atoms in a crystal lattice are derived from light scattering by using the Bragg equation, scattering from amorphous or semi-crystalline polymers is described in terms of electron density at a point in reciprocal space. The information in a SAXS profile can include d-spacing from larger spaces between lattices, but also includes scattering from any electron density seen by the X-ray beam and is described by the magnitude of the scattering wavevector.

Mithrene has a diffraction peak corresponding to the (002) crystallographic plane that appears at 4.4 nm^−1^ or at a d-spacing of 1.46 nm. This lattice spacing is observed in the SAXS regime of reciprocal space. Despite the random orientation of suspended crystallites, they will diffract to high intensity. Solution methods allow for concentrating of the early phase products, therefore the possibility of crystallites being seen via solution SAXS is much higher than that of GIWAXS, where only one reaction can be probed per thin film.

Using the quenching method as described in the experimental section, the reaction was stopped at different time points starting with 5 min, 15 min, 1 h, and then hourly until 24 h into the synthesis. Taking advantage of the high-throughput SAXS endstation (Hura et al., 2009), that allowed for rapid data collection from a large number of samples, we were able to collect data on all time points within an hour. After 15 min, a broad diffraction peak was present at 4.4 nm^−1^ in all sample SAXS profiles with sufficient sample concentration to produce adequate scattering profiles.

**FIGURE 5:**
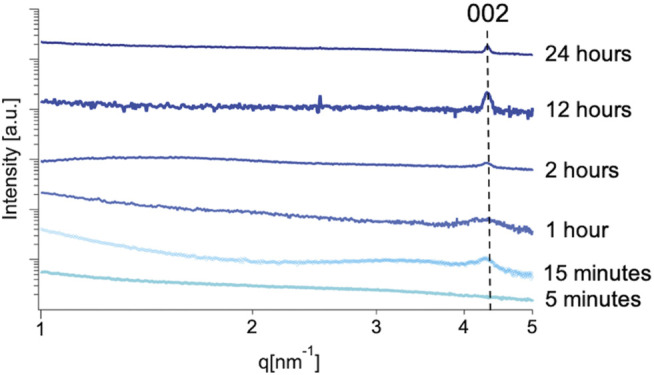
Solution SAXS profiles of early stage mithrene samples from 5 min to 24 h. Each profile has been normalized to intensity. Qualitatively, the profiles in the lower q regime differ significantly. This could be attributed to aggregation of sample in solution, as non-ideal solution was used and samples from early time scales will have less product presence. This difference could also be attributed to the size heterogeneity of the polymer product in solution.

**FIGURE 6:**
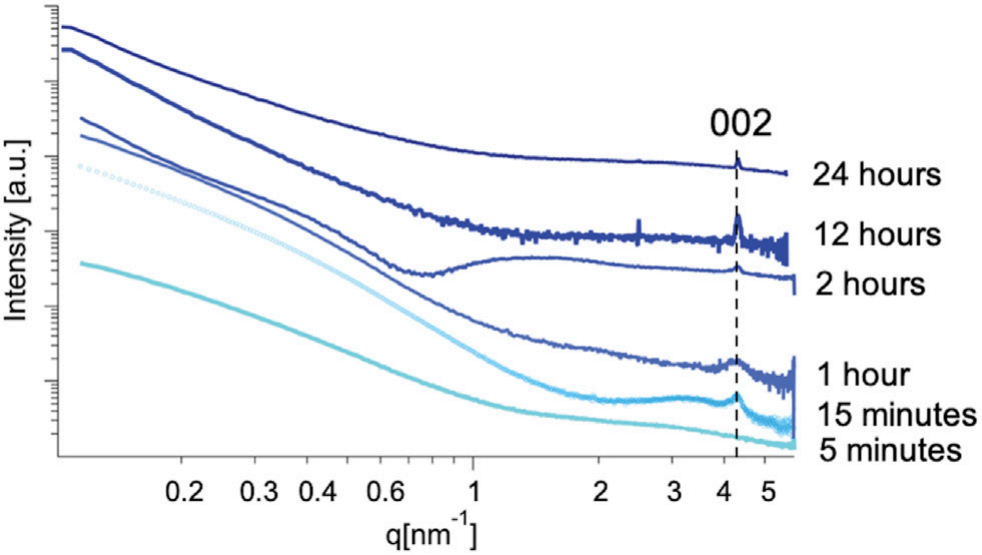
Cropped SAXS profiles of mithrene grown for 5 min, 15 min, 12-h, and 24 h. The 15-min profile shows a broad Bragg peak at 4.4 nm^−1^ which corresponds well with the known interlayer spacing of mithrene crystallites and the (002) crystallographic plane, the 24-h profile shows a sharper peak also corresponding with the (002) crystallographic plane, as well as the 12-h profile and the 5-min profile shows a complete absence of the peak.

In the case of our experiment, we were able to trace the emergence of crystalline order to <15 min using this method, where a broad peak at 4.4 nm^−1^ corresponding with the (002) crystallographic plane of the mithrene unit cell (Cuthbert et al., 2002; Schriber et al., 2018) appears in solutions from reactions quenched after 15 min of growth, but is not present in profiles from 5 min of growth, see Figures 5, 6. In each SAXS profile, we have contributions from both the amorphous polymer and the crystalline mithrene phase, thus producing a distribution of experimental intensities throughout each scattering profile. In earlier time points (Figure 5), where the mithrene (002) peak is either not present or is very broad and low intensity, the scattering profile is dominated by the amorphous polymer that can form clusters with significant size polydispersity and different spatial configurations. In order to obtain structural information from these SAXS profiles, determination of the scattering origins and developing a method for interpreting the scattering is essential. While past efforts by Vonk and Kortleve (1967), Vonk (1973) and recently, Li et al. (2019) have been successful at developing SAXS models for similar polymer systems, deconvoluting and modeling scattering contributions from both the mithrene system and the amorphous polymer is challenging and beyond the scope of this manuscript. Otherwise, overall intensity is dependent on concentration, which was not quantified during this experiment, but does not alter the location of a diffraction peak.

## Conclusion

Biphasic conditions present a difficult environment for performing *in-situ* experiments, where recreating conditions can often be plagued with environmental variables that are incompatible with standard methods. The reaction involves the organization of components into structurally well-defined aggregates (Huie, 2003). In many systems, it is both impractical and impossible to change the parameters that determine the behavior of the system and this makes studying each component’s role in the formation of the aggregates difficult (Whitesides and Grzybowski, 2002). While modeling provides the best analysis at the interface, it is also time-consuming and computationally expensive, especially when multiple phases are present. Altering solvent viscosity and the interfacial surface tension provides a tool for studying some aspects of the interfacial environment without having to resort to such methods. We determined that mithrene interfacial synthesis itself is robust but that changes to the interfacial environment will change the product yield, the reaction kinetics and the ratio between the polymer product and mithrene crystallites. The cold synthesis allowed us to identify the kinetic and thermodynamic products in the reaction and provided a useful synthetic method for isolating the two products of this reaction for individual analysis, something that had not been possible prior. The SAXS analysis provided a useful tool for observing the early-stage crystal product of the mithrene synthesis using the standard toluene-water interface. While it is difficult to quantify whether or not additional reorganization of the samples occurred between reaction quenching and placement in the X-ray beam, this was deemed unnecessary as a contrast technique like SAXS produces scattering from the most prevalent phase in the sample, and the location of the Bragg peak observed matches literature values for mithrene. The *ex-situ* approach presented that utilizes the high-throughput SAXS pipeline allows for analysis of large numbers of samples at multiple time points to be characterized. *In-situ* observation of crystal growth at the liquid-liquid interface is feasible using synchrotron sources and will be a future area of study for these systems.

## Data Availability

Publicly available datasets were analyzed in this study. This data can be found here: https://www.ccdc.cam.ac.uk/structures Cambridge Crystallographic Data Centre 186570.
