# Supplementary material for: Investigation of Nucleation and Growth at a Liquid–Liquid Interface by Solvent Exchange and Synchrotron Small-Angle X-Ray Scattering
Source: Front Chem. 2021 Jul 20;9:593637. doi: 10.3389/fchem.2021.593637 (PMC8329353; doi:10.3389/fchem.2021.593637)
Supplement: Supplementary file 1 [file DataSheet1.docx]

Supplementary Material


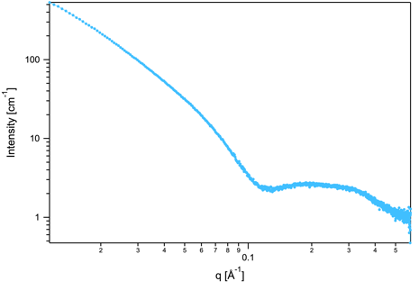


**Supplementary Figure 1**. SAXS profile of samples collected from 5 minutes of growth.


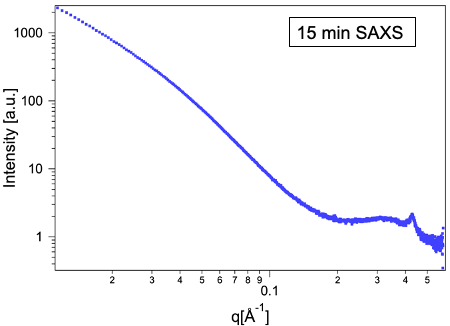


**Supplementary Figure 2.** SAXS profile of samples collected from 15 minutes of growth.


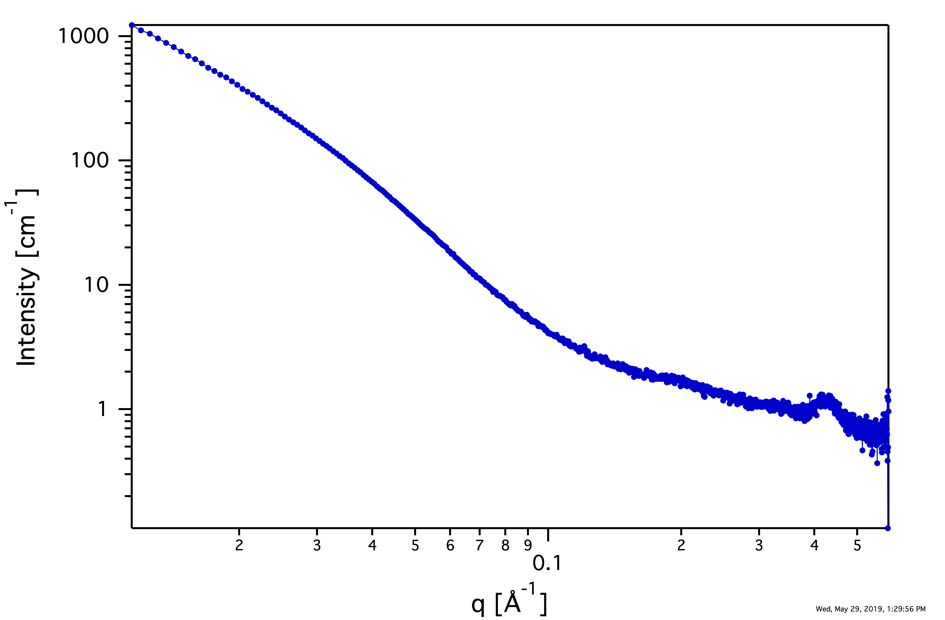


**Supplementary Figure 3**. SAXS profile from samples collected at 1 hour of growth.


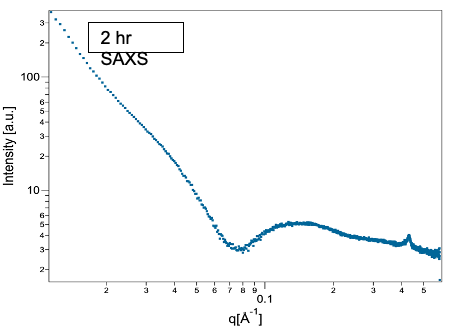


**Supplementary Figure 4**. SAXS profile from samples collected at 2 hours of growth.


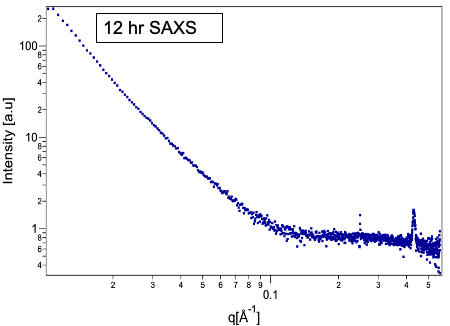


**Supplementary Figure 5**. SAXS profile from samples collected at 12 hours of growth.


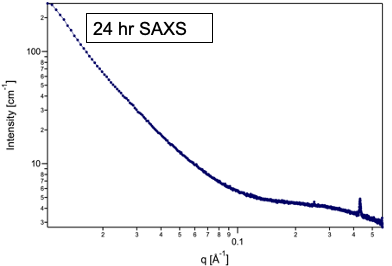


**Supplementary Figure 6**. SAXS profile from samples collected at 24 hours of growth.


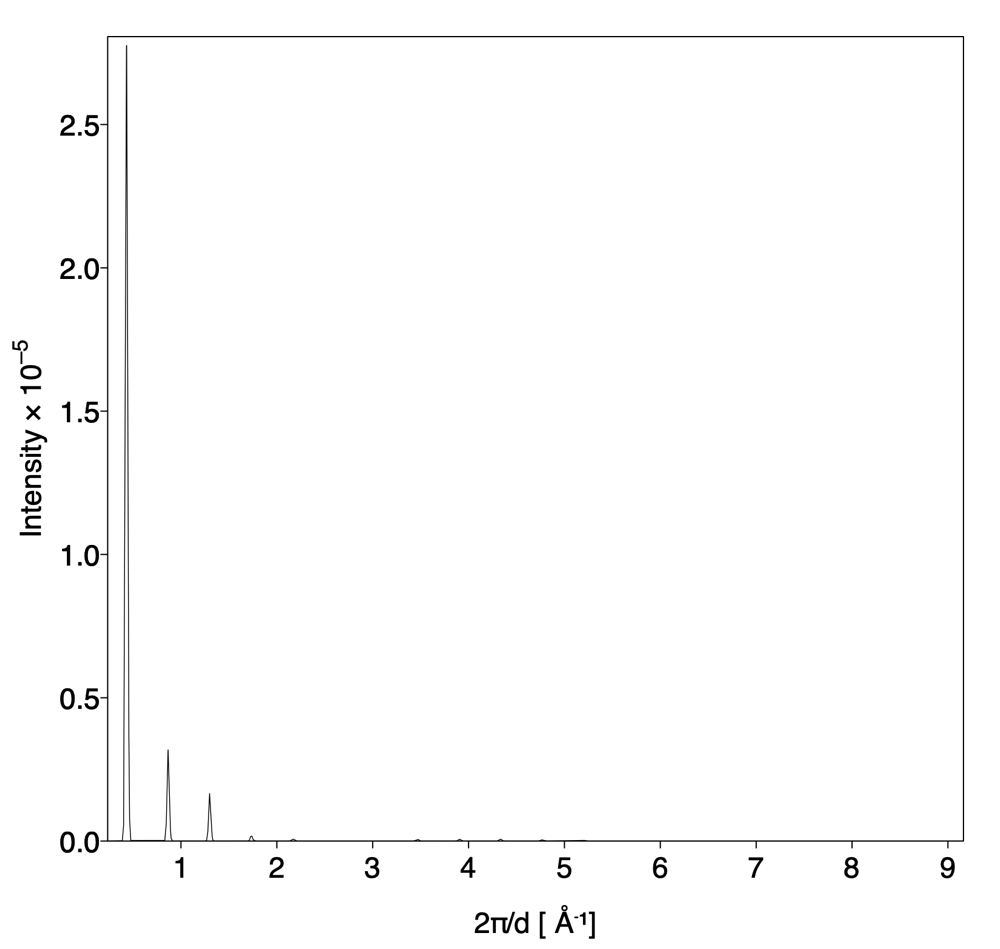


**Supplementary Figure 6**. Mithrene diffraction pattern in inverse angstroms (q), showing the (002) peak presence at 0.44 Å^-1^ or 4.4 nm^-1^.


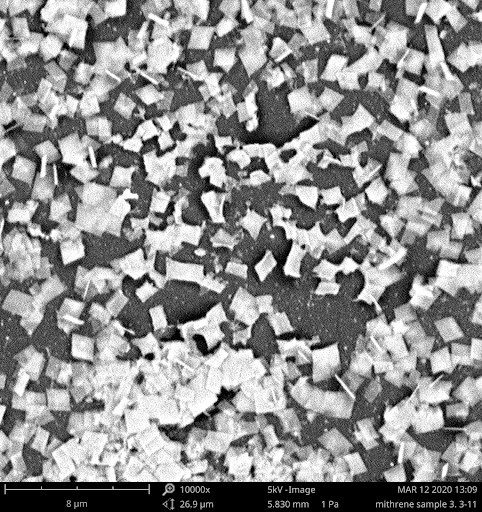


**Supplementary Figure 7**. Mithrene crystallites with presence of amorphous polymer under SEM. Showing general size polydispersity.


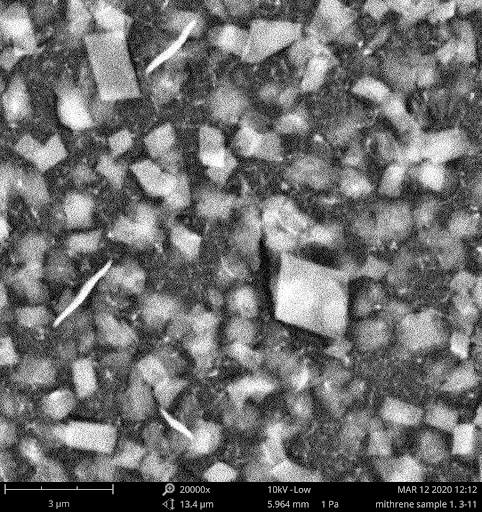


**Supplementary Figure 8**. Mithrene crystallites showing orientation both in-plane of the substrate and normal to the substrate, harvesting methods dictate how the crystallites adhere to the substrate but in-plane orientation is preferred.


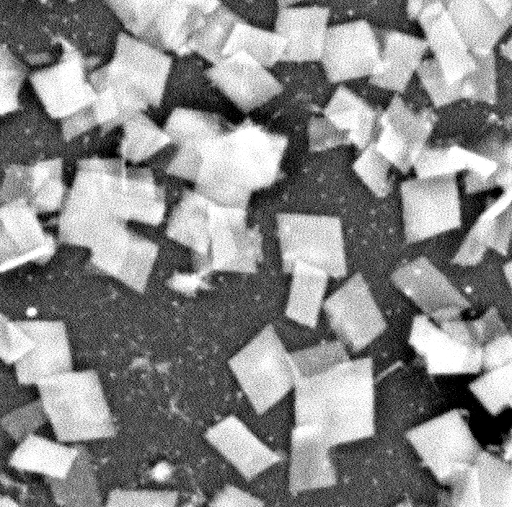


**Supplementary Figure 9**. Higher resolution image of mithrene crystallites with presence of amorphous polymer acting as a “raft” for the crystallites.
